# Dexmedetomidine Attenuates Lipopolysaccharide Induced MCP-1 Expression in Primary Astrocyte

**DOI:** 10.1155/2017/6352159

**Published:** 2017-02-14

**Authors:** Huan Liu, Jacques R. J. Davis, Zhi-Lin Wu, Amro Faez Abdelgawad

**Affiliations:** ^1^Department of Anesthesiology, Union Hospital, Tongji Medical College, Huazhong University of Science and Technology, Wuhan, China; ^2^Department of Anesthesiology, ICU and Pain Management, Benha University Hospital, Faculty of Medicine, Benha University, Benha, Egypt

## Abstract

*Background*. Neuroinflammation which presents as a possible mechanism of delirium is associated with MCP-1, an important proinflammatory factor which is expressed on astrocytes. It is known that dexmedetomidine (DEX) possesses potent anti-inflammatory properties. This study aimed to investigate the potential effects of DEX on the production of MCP-1 in lipopolysaccharide-stimulated astrocytes.* Materials and Methods*. Astrocytes were treated with LPS (10 ng/ml, 50 ng/ml, 100 ng/ml, and 1000 ng/ml), DEX (500 ng/mL), LPS (100 ng/ml), and DEX (10, 100, and 500 ng/mL) for a duration of three hours; expression levels of MCP-1 were measured by real-time PCR. The double immunofluorescence staining protocol was utilized to determine the expression of *α*2-adrenoceptors (*α*2AR) and glial fibrillary acidic protein (GFAP) on astrocytes.* Results*. Expressions of MCP-1 mRNA in astrocytes were induced dose-dependently by LPS. Administration of DEX significantly inhibited the expression of MCP-1 mRNA (*P* < 0.001). Double immunofluorescence assay showed that *α*2AR colocalize with GFAP, which indicates the expression of *α*2-adrenoceptors in astrocytes.* Conclusions*. DEX is a potent suppressor of MCP-1 in astrocytes induced with lipopolysaccharide through *α*_2_A-adrenergic receptors, which potentially explains its beneficial effects in the treatment of delirium by attenuating neuroinflammation.

## 1. Introduction

Delirium is the most common psychiatric syndrome for senior ICU patients [1], often resulting in higher mortality rates and increased duration of hospital stay [2, 3]. Its mechanism has not been completely understood yet. A line of studies indicated that neuroinflammation may play a role in its pathogenesis [4–6]. Astrocytes, the main component of glial cells in central nervous system, function not only as the basic structure skeleton, but also as a key participant in the activity of the central nervous system [7]. Immunologically they maintain a dormant state until aroused by the stimulation of pain, ischemia, or other injuries. The astrocytes will then be activated, resulting in large numbers of proinflammatory factors, leading to cytokine synthesis and resulting in neuroinflammation [8].

Of all the cytokines secreted by astrocytes, MCP-1 is the one that has been elucidated clearly. It is a potent chemotoxin and activation factor for monocytes, which can modulate the synthesis of cytokines and adhesion molecules on the cell surface, thus being an important proinflammation factor [9]. Studies showed that MCP-1 plays a pivotal role for monocyte recruitment in spinal trauma and nerve inflammation [10, 11]. Inhibiting MCP-1 secretion can dramatically attenuate the inflammation response [12]. Therefore inhibiting the expression of MCP-1 in astrocytes may provide a potential method for the treatment of nerve inflammation and delirium.

DEX is a highly selective *α*2AR agonist. A number of studies showed that it possesses anti-inflammatory capabilities [13–15]. It was found to reduce the systematic inflammatory responses in rats with endotoxemia, regulate inflammatory responses of macrophages induced by LPS [16, 17], alleviate myocardial ischemia-reperfusion injury, and reduce infarction size [18]. In the central nervous system, DEX is proven to promote neurotrophic-factor secretion in astrocytes and exerts a neuroprotective effect [19]. DEX is also a potent suppressor of lipopolysaccharide induced inflammation in activated microglial cells [20]. However, little is known regarding the effects of DEX on the inflammation of astrocytes. The following research aims to investigate the effects of DEX on the production of MCP-1 in LPS activated astrocytes and provide useful and applicable information to reveal the potential therapeutic value of DEX in the treatment of delirium in ICU patients.

## 2. Materials and Methods

### 2.1. Primary Cortical Astrocyte Culture Preparation

Primary cortical astrocytes were obtained from 1-day-old neonatal Sprague-Dawley rats. The detailed protocol was previously described [21]. Briefly, the cerebral cortices of the rats were minced with a mesh bag (100 mm). Dissociated cortical cells were then suspended in Dulbecco's modified Eagle medium containing 10% heat-inactivated fetal bovine serum and 1% penicillin/streptomycin and plated on 25 cm^2^ flasks at a density of 600,000 cells/cm^2^. Monolayers of type 1 astrocytes were obtained 6–8 days after plating. The confluent cultures were shaken at (200 rpm, 37) for 4 hours to separate the remaining microglial cells and oligodendroglial cells. The purity of primary cortical astrocytes was assessed by immunofluorescent staining for GFAP (Sigma, USA). Once established that more than 95% of the cultured cells were GFAP positive, d-cAMP (150 *μ*mlo/L) was added to induce cell maturation over a duration of 3 to 4 days.

### 2.2. Immunofluorescence Assay

Primary astrocytes were prepared from the cerebral cortex of neonatal rats as described above. Astrocytes were then cultured on cover glasses (18 mm *∗* 18 mm) placed in the 6-well plates. Dibutyryl cAMP (d-cAMP, 0.15 mM, Sigma, St. Louis, MO) was added to induce differentiation when cells attained 50%–80% confluence. Then serum-containing medium was abandoned and cover glasses were washed one time with 0.01 M PBS. The astrocytes were then fixed with 4% paraformaldehyde for 30 minutes. The slides were initially blocked with 5% goat-serum for 2 hours at room temperature and then incubated with the following two primary antibodies: *α*2A (rabbit, 1 : 50, Santa Cruz Biotechnology, Inc., Santa Cruz, CA) and GAPDH (mouse, 1 : 20,000, Millipore, Billerica, MA, USA) at 4°C overnight. After washing them with PBS three times, the slides were then incubated free from light with Cy3- or FITC-conjugated secondary antibodies (1 : 1000, Jackson ImmunoResearch, West Grove, PA, USA) for 2 hours at room temperature, rinsing well and sealing the slides with 30 *μ*l mounting medium. The stained sections were examined with a Leica fluorescence microscope, and images were captured with a CCD Spot camera. Some sections with double staining of *α*2A and GFAP were imaged with Leica SP8 Gated STED confocal microscope.

### 2.3. Experiment Protocols

In the preliminary experiment, we aimed to achieve the optimum dose of LPS for astrocyte, so cells (approximately 1,105 cells/mL) were seeded in six-well plates before being subjected to treatments. Five groups of astrocytes were subjected to various treatments. In group 1, cells were incubated in OPT for 3 hours as a control. In groups 2–5, cells were treated with 10 ng/ml, 50 ng/ml, 100 ng/ml, and 1000 ng/ml LPS; real-time PCR was used to measure MCP-1 mRNA expression. We established that astrocytes were most sensitive to 100 ng/ml LPS. Thus we utilized the optimum dose of 100 ng/ml for the following experiments. Another six groups of cells were subjected to various treatments. In group 1 cells were incubated in OPT for 3 hours as a control. In group 2, cells were treated with 100 ng/ml LPS for 3 hours; in group 3, cells were treated with 500 ng/ml DEX for 3 hours. In groups 4-5, cells were pretreated with 10 ng/ml, 100 ng/ml, and 500 ng/ml DEX for 1/2-hour and then with 100 ng/ml LPS for 3 hours.

### 2.4. Reverse Transcription and Polymerase Chain Reaction

Total RNA were prepared from astrocytes by utilizing the Trizol reagent (Invitrogen Corporation, Carlsbad, CA) according to the manufacturer's protocol. Total RNA was reversely transcribed by using Moloney murine leukemia virus reverse transcriptase (Promega, Madison, WI). Polymerase chain reaction (PCR) primers were as follows: MCP-1, sense: 5′-ACTTGACCCATAAATCTGA-3′, antisense: 5′-TGGAAGGGAALAGTGTAAT-3′; GAPDH: sense: 5′-TCC TAC CCC CAA TGT ATC CG-3′, antisense: 5′-CCT TTA GTGGGC CCT CGG-3. The following PCR condition was applied: denaturation at 95°C for 30 s, then 40 cycles of denaturation at 95°C for 5 s, annealing at 56*～*60°C for 30 s, and extension at 72°C for 30 s. The PCR products were photographed after electrophoresis through agarose gel, stained with ethidium bromide, and analyzed with Rotor-Gene Corbett software.

### 2.5. Statistical Analysis

All results observed were expressed as the means ± standard error of the mean. Intragroup comparisons were made by one way ANOVA. Binary comparisons were made by Student's *t*-test. A value of *P* < 0.05 was accepted as statistically significant.

## 3. Results

### 3.1. LPS Stimulated MCP-1 Releasing in Astrocyte

Before harvesting, morphology examined under microscope showed astrocyte adhesion on the wall, with cell body contracting, multiple cytoprocesses, and no floating. After 3 hours of coculture with LPS 10 ng/ml, 50 ng/ml, 100 ng/ml, and 1000 ng/ml, expressions of MCP-1 mRNA were measured. There was no observable difference on MCP-1 between the group containing 10 ng/ml and the group containing 50 ng/ml.

**Figure 1:**
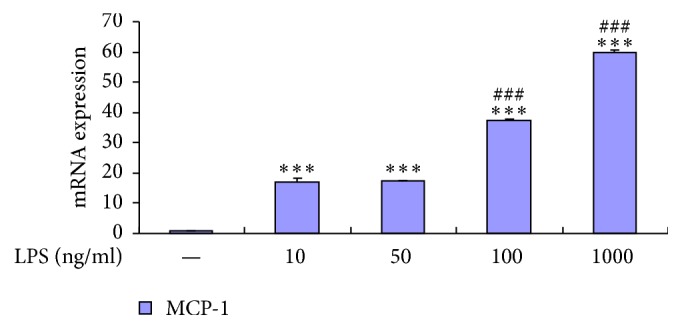
LPS dose-dependently increased stimulation of astrocytes release of MCP-1. RT-PCR shows that LPS dose-dependently increased release of MCP-1 from astrocytes. Data represent mean ± standard error of the mean of four independent experiments. ^*∗∗∗*^*P* < 0.001 compared to control group; ^###^*P* < 0.001 LPS 100 ng/ml compared to LPS 50 ng/ml group, LPS 1000 ng/ml compared to LPS 100 ng/ml group.

Consequently the expression of MCP-1 mRNA increased dose-dependently with LPS (Figure 1). Since astrocytes were most sensitive to a dose of 100 ng/ml LPS, this concentration of LPS was utilized in the following study.

### 3.2. DEX Suppresses MCP-1 Release in LPS-Stimulated Astrocytes

Expression of MCP-1 mRNA increased 37-fold in astrocytes that were being stimulated with 100 ng/ml LPS in comparison to the control group, while, in groups pretreated with DEX (10 ng/ml, 100 ng/ml, and 500 ng/ml) for 30 min, MCP-1 mRNA expression decreased dramatically under the identical stimulation of LPS.

**Figure 2:**
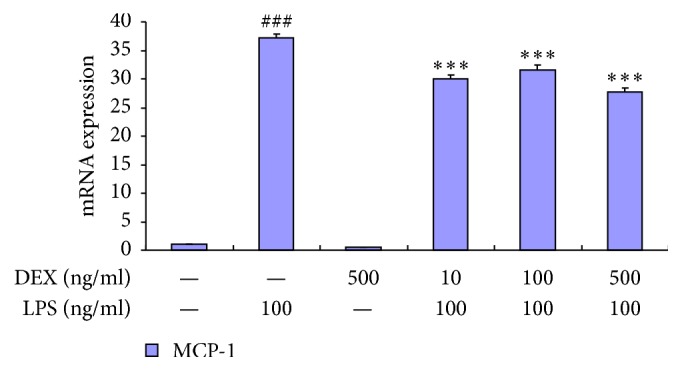
Dexmedetomidine significantly inhibited the release of MCP-1 in activated astrocytes. RT-PCR shows that dexmedetomidine significantly inhibited the release of MCP-1 in activated astrocytes. Data represent mean ± standard error of the mean of four independent experiments. ^###^*P* < 0.001 versus control group; ^*∗∗∗*^*P* < 0.001 versus LPS 100 ng/ml group.

To exclude the possible toxic effects of high concentration DEX, maturely differentiated astrocytes were pretreated with 500 ng/ml DEX alone for 3 hours. The expression of MCP-1 mRNA was then observed to remain the same as the control group. This suggested that DEX possessed no poisonous effect on astrocytes (Figure 2).

### 3.3. DEX Suppresses the MCP-1 Releasing through *α*2AR

**Figure 3:**
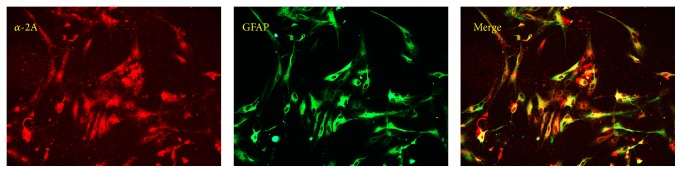
Double immunofluorescence exhibited that *α*2A-adrenoreceptors (*α*2A-R) colocalize with (a) glial fibrillary acidic proteins (GFAP; an astrocytic marker). Scale bar = 20 *μ*m.

Double immunofluorescence displayed that *α*2AR colocalize with GFAP, which evidently proved the existence of *α*2AR in astrocytes (Figure 3). As a specific agonist of *α*2AR, DEX might potentially suppress the release of MCP-1 through this pathway.

## 4. Discussion

This is the first study to demonstrate that DEX was capable of attenuating the expression of MCP-1 mRNA, a potent proinflammatory factor in primary cultured astrocytes activated by LPS. This provides additional evidence for DEX's therapeutic value in neuroinflammation and related illnesses such as delirium.

Astrocytes are the chief components of glial cells in the central nervous system and maintain a key role in the process of neuroprotection against various kinds of injuries [22, 23]. They specially buffer ions like K^+^, scavenge free radicals for the maintenance of the extracellular environment, provide substrates for neurotransmitters and antioxidants, secrete neurotrophic factors, promote much needed neovascularization, and assist in the regeneration of synapses and neurons [24–28]. Astrocytes can be activated by inflammatory mediators in a wide range of CNS pathologies, including ischemia, infection, and neurotrauma [29–31]. Through the release of numerous proinflammatory cytokines and chemokines, astrocytes are thereby involved in the process of immune response initiation and regulation [32]. Astrocytes activation is thought to be protective via destruction of pathogens, removal of debris, and promotion of tissue repair [33]. However, prolonged and sustained inflammation may have cytotoxic effects, aggravating the incidence and the severity of the disease. Symptoms such as fever, somnolence, hyperalgia, and allodynia are all related to the neuroinflammation process [34].

Of the chemokines, which astrocytes released, MCP-1 is the one that has been clarified quite clearly. It is produced by monocytes, fibroblast cells, B cells, and endothelium and glial cells. In the central nervous system, the astrocytes are the main source [35]. It chemoattracts and activates monocytes, modulates cytokines and adhesion molecule production, and initiates inflammatory responses [36]. MCP-1 receptors on the surface of astrocytes modulate the production with regard to a positive feedback mechanism. MCP-1 could possibly be involved in secondary inflammatory response in the site of spinal injury [37]. It has been observed to cause neuronal death via direct apoptotic mechanisms through specific cell surface chemokine receptors [38]. MCP-1 receptor antagonists dramatically controlled the inflammation response and enhanced neurological recovery in rats with spinal-cord contusion injuries [10]. With administration of MCP-1-antibody, cases of neuropathologic pain in sciatic and L5 spinal nerve ligation models can be significantly alleviated [12].

Nonselective alpha adrenergic receptor agonists such as noradrenaline can reduce the production of MCP-1 in rat-cultured astrocytes in the presence of an inflammatory stimulus [39], such as LPS. The effect was reversed by alpha-2 adrenergic receptor antagonist Yohimbine which indicates alpha-2 adrenergic receptors are important cell signaling pathways for modulating the release of inflammatory cytokines [40].

DEX, an alpha-2 agonist, is a widely used sedative in intensive care units. Numerous studies revealed that, other than its sedation effect, DEX possesses potent anti-inflammation capabilities as well as being greatly beneficial as a neuroprotective agent [41]. The aim of this research was to investigate the effect of DEX on the production of MCP-1 in primary cultured astrocytes activated by LPS. LPS is a potent stimulus which induces a process of an inflammation response in endotoxemia. Firstly we studied the most optimum dose of LPS for stimulating the release of MCP-1 in astrocyte. We used 50 ng/ml, 100 ng/ml, and 1000 ng/ml LPS in this case for the stimulation of astrocytes and found that, with a dose of 100 ng/ml LPS, stimulation of astrocytes displayed the most sensitive reaction in regard to the release of MCP-1. After establishing the correct and most applicable dose of LPS, we pretreated astrocytes with 10 ng/ml, 50 ng/ml, and 500 ng/ml dexmedetomidine and then with 100 ng/ml LPS stimulation. All groups showed that there was a decrease in the rate of synthesis of MCP-1 RNA. To exclude the toxic effects of DEX regarding astrocytes, astrocytes were treated with a high dose of DEX alone. Results indicated that there was no observable difference compared to the control group. This suggested that DEX possesses no toxic effect on astrocytes.

DEX's capability of organ protection has been confirmed by numerous studies. DEX inhibits cell apoptosis and death and increases the survival rate of neurons by modulating the release of catecholamine and glutamate in the central nervous system [42]. Research indicated that DEX can increase survival rate of rats diseased by septic shock in manner of decreasing systematic inflammation [16]. DEX suppresses the release of cytokines IL-1*β*, TNF-*α*, and IL-6 and promotes the recovery of spinal injury in rats [15]. Besides its anti-inflammation capability, DEX promotes the release of glial cell line-derived neurotrophic factors in rat astrocytes, for neuronal and synapse regeneration [43]. DEX has no direct cytoprotective effect on H_2_O_2_ exposed neurons; however clinically relevant concentrations of DEX are neuroprotective against oxidative damage due to the stimulation of astrocytic-adrenoceptors, causing release of HB-EGF which in turn activates neuronal EGF receptors [44].

However, it still remains unclear whether DEX has a direct effect on anti-inflammation of astrocytes or not. In our research, primary cultured astrocytes were pretreated with different doses of DEX and then stimulated with 100 ng/ml LPS; real-time PCR indicated that the following concentrations: 10, 100, and 500 ng/ml DEX, dramatically inhibited the release of MCP-1 mRNA. Thus, these results indicate that DEX possesses the capability of inhibiting the production of MCP-1 on astrocytes.

There are however some limits within our study. Firstly, the clinical therapeutic plasma concentration of DEX is 0.3–2 ng/ml; research suggests that 1 ng/ml DEX has no observable effect on the microglial cell inflammatory response. Our research suggested that 10 ng/ml DEX inhibits the release of MCP-1. Whether a lower dose of DEX could possess the same effect still remains to be known. High concentrations of DEX can increase free cytosolic calcium concentration in astrocytes. The effect is partly blocked by Yohimbine and by Idazoxan, an inhibitor at the imidazoline-preference site [45]. The molecular signaling pathway of DEX regarding the production of MCP-1 in LPS-stimulated astrocytes is yet to be investigated.

In summary, this study indicates that DEX possesses the potential to decrease inflammatory response by inhibiting MCP-1 expression through *α*2AR on astrocytes and in addition exerts a protective effect on neuroinflammation. This research provides applicable data and results which indicate the potential value of DEX in the treatment of delirium in ICU patients.
